# Citrate and calcium kidney stones

**DOI:** 10.1093/ckj/sfaf244

**Published:** 2025-08-04

**Authors:** Alireza Zomorodian, Orson W Moe

**Affiliations:** Charles and Jane Pak Center for Mineral Metabolism and Clinical Research, University of Texas Southwestern Medical Center, Dallas, TX, USA; Charles and Jane Pak Center for Mineral Metabolism and Clinical Research, University of Texas Southwestern Medical Center, Dallas, TX, USA; Department of Internal Medicine, University of Texas Southwestern Medical Center, Dallas, TX, USA; Department of Physiology, University of Texas Southwestern Medical Center, Dallas, TX, USA

**Keywords:** acid–base homeostasis, calcium nephrolithiasis, citrate metabolism, hypocitraturia

## Abstract

Citrate, a tricarboxylic acid cycle intermediate, plays a central role in renal physiology by acting as both a urinary base equivalent and a potent inhibitor of calcium stone formation. Hypocitraturia, a common metabolic abnormality in calcium nephrolithiasis, is not a binary disorder but a continuum shaped by acid–base status, diet, potassium balance, proximal tubular handling, and systemic citrate status.

We provide an update on the biology of citrate, renal regulation of its excretion, clinical pathophysiology, and treatment of hypocitraturia. Identical urinary citrate levels may have different implications depending on systemic acid–base status and urinary calcium excretion. Hypocitraturia prevalence is increasing, paralleling rises in metabolic syndrome, obesity, and dietary habit changes. Experimental models confirm that systemic or intracellular acidosis, potassium deficiency, and upregulation of renal transport and metabolism of citrate reduce urinary citrate, enhancing stone risk.

Potassium citrate remains the cornerstone of therapy, increasing both urinary citrate and pH. However, its use requires caution in calcium phosphate stone formers and patients with chronic kidney disease. Citrate resistance, defined as inadequate urinary citrate response despite good potassium delivery, is a therapeutic challenge. Novel interventions including sodium-dicarboxylate cotransporter-1 (NaDC-1) inhibitors and citrate analogs such as hydroxycitrate may offer future alternatives.

In conclusion, urinary citrate must be interpreted within physiological and clinical contexts. Recognizing hypocitraturia as a modifiable, non-binary risk factor allows for more precise risk stratification and individualized therapy in stone prevention, particularly when lithogenicity overlaps with acid–base and renal abnormalities.

## CITRATE BIOLOGY

**Figure 1:**
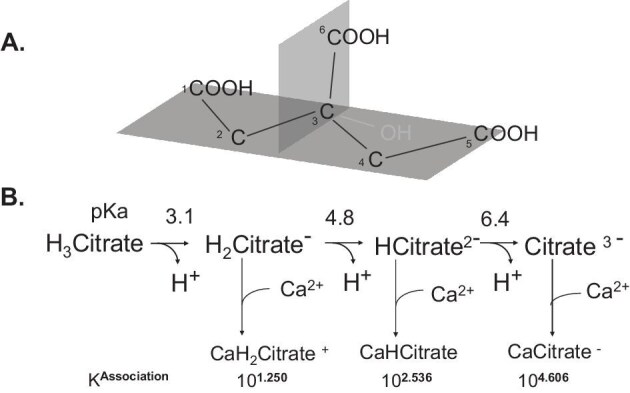
Citrate structure, polyvalent anion states, and calcium chelation. (**a**) Citrate's carbon backbone (C1–C5) is arranged in a planar configuration, with the carboxyl group (C6) and the hydroxyl group on C3 oriented in a perpendicular plane. (**b**) Citric acid undergoes stepwise dissociation of three protons (H⁺) to form mono-, di-, and trivalent citrate anions. The corresponding p*K*_a_ values for each dissociation step are indicated. Citrate anions in all valence states can bind calcium (Ca²⁺), forming soluble Ca²⁺–citrate complexes, with the highest binding affinity observed for the Ca²⁺citrate³⁻ complex. The calcium association constants (*K*^association^) are also shown.

Citrate is a six-carbon tricarboxylic acid (Fig. 1a) which is an intermediate in several metabolic and synthetic pathways. It is the “first” compound in the tricarboxylic acid (TCA) cycle. In the appropriate ambient pH, citrate can exist as neutral, mono-, di-, or tricarboxylic states (Fig. 1b). Human plasma citrate concentration is usually 100–150 μM, with a certain fraction complexed with plasma proteins and cations [1]. Despite its relatively low concentration, citrate plays diverse biological roles across various organs and body fluids.

In mammalian urine where it is present in millimolar concentrations, citrate has two main roles: **1**. Acts as a base equivalent, allowing alkali to be excreted in urine without a dangerous rise in urine pH, and **2**. A calcium chelator that inhibits calcium crystal nucleation, growth, and aggregation; thus, it is a crucial suppressor of kidney stone formation [2].

**Figure 2:**
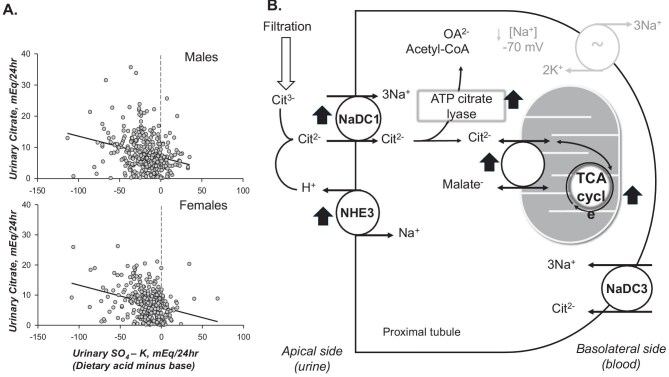
Citrate and acid–base status. (**a**) Relationship between urinary citrate excretion and net dietary acid load, calculated as urinary sulfate (a surrogate of dietary acid intake) minus urinary potassium (a surrogate of dietary alkali). Data are from male and female kidney stone formers in the Mineral Metabolism Clinic at UT Southwestern Medical Center (1972–2015). Some participans were newly evaluated with baseline 24-hour urine collections, while others were already receiving potassium citrate (K₃citrate) therapy. (**b**) Cellular model of citrate handling in the renal proximal tubule [61]. Urinary citrate excretion is determined by the filtered load, which is the ultrafilterable plasma citrate concentration multiplied by the glomerular filtration rate, minus proximal tubular reabsorption. See the main text for a detailed description of transporters, metabolic pathways, and regulation during acid loading.

Citrate is freely filtered at the glomerulus and exclusively reabsorbed in the proximal tubule. In hypocitraturic conditions such as acid loading (Fig. 2a), several parallel adaptations transpire in unison to reduce urinary citrate thus conserving and preventing urinary base loss (Fig. 2b). An unintended consequence of physiologic hypocitraturia to conserve alkali is diminished capacity to chelate calcium in urine.

### What is hypocitraturia?

An important concept is that citraturia is not a dichotomous variable. Interpretation of urinary citrate levels is beyond just recognizing whether the value is outside the normal range of the clinical reference laboratory. As stated before, citrate has dual roles in the urine. In the context of kidney stone risk, these dual roles may function in conflict. Hypocitraturia is often defined by clinical laboratories as urinary citrate excretion <320 mg or 1.66 mmol/day. Hypocitraturia is a common metabolic abnormality in patients with calcium nephrolithiasis [3]. Knowing that the cut-off for clinical “hypocitraturia” is arbitrary, one should never interpret citraturia as a dichotomous variable. There are two questions one should consider when appraising citraturia.

The first question is whether citraturia is physiologically appropriate. If accompanied by high urinary potassium (e.g. >60 mEq/day, indicative of high dietary alkali intake), urinary citrate of 1.66 mmol (320 mg)/day is actually abnormally low suggestive of deranged tubular function (tubular hyperabsorption from any cause) or systemic abnormality. In a similar argument, urinary citrate of 1.66 mmol/day is considered “high” if markers of acid intake (urinary NH_4_^+^, SO_4_^2−^, urea) are elevated and compensatory hypocitraturia is appropriate and expected. Fundamental biologic principles dictate that if a parameter is expected to be high or low under specific physiologic conditions, but instead it falls within the “normal range”, this signifies “abnormality”. Figure 2a shows the linear relationship of urinary citrate to a surrogate of net acid ingestion (sulfate–potassium). Figure 2b illustrates the cellular model of citrate handling in the renal proximal tubule. Filtered citrate, primarily in its trivalent form (citrate³⁻), is converted to the divalent form (citrate²⁻) via luminal H^+^ titration by the apical Na⁺/H⁺ exchanger (NHE3), allowing uptake through the sodium-dicarboxylate cotransporter 1 (NaDC-1). Intracellular citrate has two fates: [1] a cytoplasmic pathway, serving as a carbon source for processes such as fatty acid synthesis, or [2] a mitochondrial pathway, entering the TCA cycle in exchange for malate to support ATP production. Additionally, basolateral NaDC-3 imports citrate from the circulation, underscoring the proximal tubule's central role in citrate metabolism. During acid loading, coordinated upregulation of transport and metabolic pathways enhances citrate reabsorption and utilization (black arrows), thereby reducing urinary citrate excretion.

An alternative, more pragmatic approach is to interpret the absolute urinary citrate concentration primarily as a marker of calcium chelation capacity, irrespective of the underlying mechanisms leading to that value or its appropriateness in the context of acid–base status. However, even with this approach, several circumstantial variables must be considered, including urinary calcium, oxalate, phosphate, pH, and volume, all of which influence stone risk and citrate's protective role. Urinary citrate of 320 mg (1.66 mmol/day) clearly contributes to stone risk if urinary calcium is 240 mg (6 mmol/day) whereas the same citrate excretion rate with urinary calcium of 120 mg (3 mmol/day) does not pose that much of a stone risk.

In summary, interpretation of urinary citrate in clinical testing is beyond just labeling whether it is normal or abnormal. Citraturia is not a dichotomous variable, thus requires some effort in comprehending physiology and pathophysiology. We will consolidate current clinical data on the role of hypocitraturia in calcium kidney stone formation, including its epidemiology, evidence from preclinical studies, and underlying etiological factors.

#### Epidemiology

Although there is no universally established cut-off, an arbitrary threshold of 1.66 mmol (320 mg)/day is often used to define hypocitraturia, which has been reported in 20% to 60% of patients with calcium kidney stones [4–6]. A retrospective analysis compared metabolic profiles across two time periods (1988–1994 vs 2007–2010) and found a significant increase in hypocitraturia prevalence among stone formers, from 46% to 60% [7]. This trend paralleled broader shifts in morbidities, including increased rates of obesity, diabetes, and metabolic syndrome [7]. Notably, the prevalence of stone disease overall has increased in the general population, from 4% in 1976–1980 to 9% by 2007–2010 in the USA, with a relative rise in percentage in female patients [8].

Dietary habits significantly influence urinary citrate levels. Withdrawal of fruits and vegetables for just 14 days in healthy individuals led to a 44% reduction in urinary citrate excretion, whereas reintroduction of a diet rich in fruits and vegetables increased urinary citrate by 68% in hypocitraturic stone formers [9]. These findings underscore that hypocitraturia is not only common but also modifiable, with dietary patterns playing a major role in its prevalence and in stone formation risk.

In a large national cohort of veterans with a history of kidney stones, Ganesan *et al.* reported that nearly 20% of patients had hypocitraturia <1.04 mmol (200 mg)/day, highlighting the burden of this abnormality in contemporary stone formers [10]. Features of metabolic syndrome, including obesity and insulin resistance, are significantly associated with lower urinary citrate excretion, contributing to an increased risk of calcium kidney stone formation [11, 12]. Together, these data suggest that hypocitraturia is a prevalent and increasingly recognized metabolic defect in the stone-forming populations, driven by changing dietary habits, the rising burden of metabolic diseases, and broader epidemiologic transitions.

## PRECLINICAL MODELS

Preclinical models have significantly advanced our understanding of hypocitraturia in the pathogenesis of kidney stone disease. Although genetically hypercalciuric rats naturally exhibit hypocitraturia and are predisposed to calcium nephrolithiasis even without acid loading, induction of acid loading further reduces urinary citrate excretion, increasing urinary supersaturation and stone formation risk [13, 14].

Urinary citrate excretion is regulated by the activity of the sodium-dicarboxylate cotransporter 1 (NaDC-1), in the apical membrane of renal proximal tubular cells, encoded by the *SLC13A2* gene. Alterations in NaDC-1 activity directly influence citrate reabsorption and consequently, urinary citrate levels [15, 16]. Increased activity or expression of NaDC-1 leads to enhanced citrate reabsorption, resulting in decreased urinary citrate excretion promoting calcium stone formation [17, 18].

In vitro studies and animal experiments have confirmed that both systemic and renal tubular acidosis enhance proximal tubular citrate reabsorption, which must be coupled with enhanced citrate metabolism within proximal tubular cells [18, 19] (Fig. 2b). Potassium depletion similarly induces intracellular acidosis in tubular cells, which enhances citrate reabsorption and reduces urinary citrate levels [20]. As a result, the urinary environment becomes supersaturated with calcium salts due to the loss of citrate's inhibitory effects on calcium crystallization, promoting nucleation, aggregation, and crystal retention [21].

Collectively, preclinical models underscore that hypocitraturia is not merely an associated finding but a causative factor in the development of calcium-based nephrolithiasis, mediated through multiple metabolic and transport pathways.

## CAUSES OF HYPOCITRATURIA

Hypocitraturia can arise from a range of physiological, dietary, pharmacologic, and pathologic conditions that influence systemic acid–base, renal handling of citrate, or both [2]. The primary mechanisms involve enhanced proximal tubular citrate reabsorption, increased cytoplasmic and/or mitochondrial uptake and metabolism, or action together, in response to systemic or intracellular acidosis, volume depletion, or potassium loss [2, 17, 22] (Fig. 2b).

### Metabolic acidosis

Systemic acidosis, whether overt or subclinical, is a well-established driver of hypocitraturia. Acidosis promotes reabsorption of citrate in the proximal tubule via upregulation of NaDC-1 transporter, cytoplasmic metabolism by ATP citrate lyase, and uptake and metabolism by mitochondria; all resulting in reducing urinary citrate excretion [23]. Conditions such as chronic diarrhea, complete or incomplete distal renal tubular acidosis (idRTA), and high dietary acid load (e.g. protein) suppress citrate excretion regardless of whether systemic pH is lowered [19, 24–26].

### Potassium depletion

Potassium depletion regardless of whether there is hypokalemia, leads to intracellular acidification of proximal tubular cells, which similarly enhances NaDC-1 mediated citrate reabsorption [27]. This mechanism links potassium depletion, whether due to gastrointestinal losses (e.g. diarrhea) or urinary loss (e.g. kaliuretic diuretics), with hypocitraturia.

### Dietary factors

Diets low in fruits and vegetables, which are the key sources of potassium alkali, are associated with lower urinary citrate [28]. By contrast, diets high in animal protein increase acid production and lower urine citrate levels [24] (Fig. 2a). Controlled feeding studies have demonstrated that dietary interventions can significantly modulate urinary citrate excretion [29].

### Gastrointestinal disorders

Malabsorptive states such as chronic diarrhea, inflammatory bowel disease and bariatric surgery (especially Roux-en-Y gastric bypass) contribute to hypocitraturia through intestinal bicarbonate and potassium loss, and volume depletion [30, 31].

### Medications

Several drugs are associated with hypocitraturia. Carbonic anhydrase inhibitors (e.g. acetazolamide, topiramate) directly reduce citrate excretion by inducing proximal renal tubular acidosis [32]. Thiazide diuretics and loop diuretics may lower citrate indirectly by causing hypokalemia [33]. Angiotensin-converting enzyme inhibitors such as enalapril can also reduce urinary citrate excretion by enhancing renal proximal tubular ATP citrate lyase activity and intracellular pH and citrate metabolism, independent of systemic acid–base changes [34].

### Obesity and insulin resistance

There is evidence linking obesity and insulin resistance to reduced urinary citrate [12]. Obese individuals may have greater dietary acid intake or increased endogenous acid production, leading to higher acid load on the kidney. Although net acid excretion rises to compensate, insulin resistance impairs renal ammoniagenesis, resulting in lower urinary pH and promoting hypocitraturia through enhanced proximal tubular citrate reabsorption. [35, 36]. Insulin resistance has been associated with a defect in ammonium (NH_4_^+^) production and excretion leading to acidic urine pH [37]. This acidic environment enhances proximal tubular citrate reabsorption, thereby reducing urinary citrate levels and contributing to hypocitraturia [2].

### Genetic and other idiopathic causes

In some patients, hypocitraturia occurs in the absence of identifiable systemic or local abnormalities, suggesting potential genetic differences in citrate metabolism or transporter regulation, or yet unidentified acquired causes. Previous study investigating the association between NaDC-1 gene polymorphisms and urinary citrate excretion reported that certain single nucleotide polymorphisms in the *SLC13A2* gene were associated with variability in urinary citrate levels among patients with calcium stones [38]. These findings suggest but not prove potential genetic contribution to idiopathic hypocitraturia via altered transporter activity. However functional sequelae of these polymorphic alleles have not been determined.

## INTERVENTIONS TO CORRECT HYPOCITRATURIA

Other than removing the underlying cause(s), alkali therapy is the cornerstone of treatment for hypocitraturia. The goal of therapy is to reduce the urinary supersaturation of calcium salts and prevent stone recurrence. In rats fed a high-casein (acid-containing protein) diet, the acid and bone biochemical abnormalities can be completely abrogated by neutralization of the acid load using citrate indicating that the acid component from the casein is responsible for the bone resorption [39, 40]. A range of alkali formulations are available, with varying mechanisms of action, dosing regimens, side effect profiles, and contraindications (Table 1). Although citrate is a key intermediate in energy metabolism, current evidence does not indicate adverse metabolic effects from high-dose citrate supplementation; however, long-term safety is not officially documented [41].

**Table 1:** Formulations of alkali therapies for hypocitraturia.

Therapy	Dosage form	Typical dosage (adults)	Side-effects	Consideration
potassium citrate	oral tablet, extended release (10 mEq; 15 mEq; 5 mEq)	30–60 mEq/day in divided doses, maximum dose: 100 mEq/day	GI upset, hyperkalemia	renal insufficiency (eGFR <45 ml/min/1.73 m²), hyperkalemia
sodium bicarbonate	oral tablet (325 mg; 650 mg)	Start with 48 mEq (4 g), then continue with 12–24 mEq (1–2 g) every 4 hours, maximum dose: 186 mEq/day (if age <60 y/o), or 93 mEq/day (if age >60 y/o)	dry mouth, diuresis edema, GI discomfort	heart failure, hypertension, sodium-sensitive conditions such as cirrhosis, kidney dysfunction
magnesium citrate	oral capsule (100, 125 133.3 mg), oral liquid (1.745 g/30 mL)	42 mEq K, 21 mEq Mg and 63 mEq of citrate per day	diarrhea, hypermagnesemia in renal impairment	severe renal impairment
potassium bicarbonate	oral tablet, effervescent (10 mEq; 20 mEq; 25 mEq)	10–20 mEq 2 to 4 times daily	GI upset, hyperkalemia	renal insufficiency, hyperkalemia
citric acid, potassium citrate, and sodium citrate (polycitra)	334–550–500 mg/5 ml	15 to 30 ml/day	nausea, vomiting, diarrhea	ESRD, untreated Addison's disease, heart failure
citric acid and sodium citrate (bicitra)	oral solution, 334 mg-500 mg/5 ml; 640 mg-490 mg/5 ml	10 to 30 ml 4 times daily	nausea, vomiting, diarrhea	same as sodium-based therapies

### Mechanism of action

Alkali agents have two effects on urinary chemistry: (i) increase urinary citrate excretion and (ii) alkalinize the urine. Citrate salts such as potassium citrate and sodium citrate are metabolized to CO_2_ and H_2_O in the liver, a process that consumes H^+^ equivalents from metabolic CO_2_ and generates HCO_3_^−^ as a metabolic byproduct, thus providing an alkali load. This is how citrate provides a systemic alkali load [42]. A good illustrative example is that ingestion of K_3_Citrate (potassium citrate) leads to citraturia but ingestion of an equivalent amount of H_3_Citrate (citric acid) does not lead to any change in urinary citrate because ingestion of citric acid provides protons that counteract the base generated by citrate metabolism resulting in a net-neutral effect [43]. Systemic alkalinization reduces renal tubular reabsorption of citrate, thereby increasing its excretion in the urine [44]. Additionally, the resulting increase in urinary pH inhibits calcium oxalate (to a small extent) and uric acid (to a large extent) stone formation by reducing supersaturation [45, 46].

Multiple clinical trials have demonstrated efficacy of alkali therapy (Table 2). Potassium-magnesium citrate increased urinary citrate levels and pH, resulting in significantly fewer stone recurrences [47]. Barcelo *et al.* also found that potassium citrate not only elevated urinary citrate but also consistently raised urinary pH into a range associated with reduced calcium salt crystallization [48]. Xue *et al.* showed that sodium bicarbonate increases urinary pH, although its citrate-raising effect was less robust than that of citrate-containing alkali, possibly due to the sodium load [49]. Unlike sodium chloride, sodium bicarbonate does not significantly increase urinary calcium excretion possibly due to the opposing effects of alkali and sodium loads, making it a safer alkali option for calcium stone formers as well as associated conditions such as hypertension or heart failure risk [50].

**Table 2:** Summary of clinical trials evaluating potassium citrate (KCit) in stone prevention.

Study	Duration	Subjects (KCit/Control)	Remission Rate (% KCit/Control)
Pak, 1983 [46]	3 years	53/—	90/—
Pak, 1985 [57]	1–4 years	89/—	80/—
Pak, 1986 [58]	2 years	37/—	90/—
Barcelo, 1993 [48]	36 months	18/20	72/20
Hofbauer,1994 [59]^a^	36 months	36/12	31/27
Ettinger, 1997[47]^b^	37 months	48/24	87/37
Soygur, 2002[60]	12 months	28/28	100/71

a Used potassium-sodium citrate combination

b Used potassium-magnesium citrate

Potassium citrate is preferred over sodium citrate formulations for stone prevention. It not only provides an alkali load that increases urinary citrate excretion but also reduces urinary calcium excretion. Potassium promotes proximal tubular intracellular alkalinization, decreasing citrate reabsorption and thus raising urinary citrate more effectively than sodium. In contrast, sodium-based formulations can increase urinary calcium excretion due to the proximal tubule effect, potentially offsetting their benefits in calcium stone formers. Therefore, the choice of citrate formulation should consider both its effect on urinary citrate and its impact on calcium excretion, particularly in patients with hypercalciuria.

Overall, alkali therapy corrects the acid–base imbalance (regardless of whether there is overt clinical acidosis) and decreases the risk of stone formation. The diverse forms of available alkali therapies provide clinicians with a range of options to tailor treatment based on patient-specific factors such as comorbidities, tolerance, cost, and personal preferences (Table 1). Among these, potassium citrate remains the most extensively studied and widely used therapy due to its dual benefit of supplying both citrate and potassium as some patients are also on thiazide therapy. However, in patients with chronic kidney disease or at risk for hyperkalemia, sodium-based or magnesium-containing alternatives may be preferable.

Magnesium-based formulations such as magnesium potassium citrate may offer added benefits by contributing to the inhibition of calcium oxalate crystallization, although they are less studied and not routinely used as first-line agents. Similarly, polycitra solution and Bicitra offer palatable liquid formulations useful in pediatric populations or those with pill aversion but carry higher sodium content, limiting their use in salt-sensitive patients. Individualized therapy, combined with dietary modifications and careful metabolic monitoring, offers the best outcomes. Increasing dietary alkali through greater intake of fruits and vegetables may offer synergistic benefits and enhance long-term adherence to achieving treatment goals, including urinary citrate levels above 1.66 mmol (320 mg)/day and urinary pH between 6.0 and 7.0 (Table 3). However, it is important to consider that this 1.66 mmol (320 mg)/day cut-off is arbitrary and does not account for physiologic context. Rather than applying a strict threshold, urinary citrate should be interpreted relative to factors such as net acid excretion and urinary calcium. For example, a citrate-to-net-acid-excretion ratio or citrate-to-calcium ratio may better reflect stone risk and guide management decisions. Thus, while achieving citrate excretion above 1.66 mmol (320 mg)/day remains a practical clinical target, interpretation should be individualized based on the patient's acid–base status and overall lithogenic risk profile.

**Table 3:** Stepwise approach to evaluation and management of hypocitraturia.

Step	Evaluation/intervention	Rationale
1. Clinical hypocitraturia	24-hour urine citrate (<1.66 mmol (320 mg)/day commonly used as arbitrary cut-off in reference labs	Establish clinical diagnosis and baseline
2. Identify comorbid stone risks in 24 hr urine	Hypercalciuria, hyperoxaluria, hyperuricosuria, low urine volume, urine pH	The impact of hypocitraturia on lithogenicity depends on other comorbid stone risk factors
3. Assess potassium and acid–base status	Check serum potassium and bicarbonate and urinary markers of alkali load (K), and acid load (NH₄⁺, sulfate)	Identify systemic and/or potassium status contributing to hypocitraturia
4. Evaluate dietary habits	Fruit/vegetable intake, animal protein consumption	Low alkali or high acid diets reduce urine citrate
5. Review potential etiology of hypocitraturia including medications	Gastrointestinal alkali losses, complete or incomplete distal renal tubular acidosis, chronic kidney disease, diuretic use loops, carbonic anhydrase inhibitors,	Diagnose and treat underlying conditions that predisposes to hypocitraturia
5. Trial of dietary intervention	Increase intake of fruits and vegetables; reduce excessive animal protein intake	Enhances citrate excretion and reduces acid load which also reduces calciuria
6. Pharmacologic alkali supplement	Start potassium citrate unless contraindicated; consider sodium bicarbonate or magnesium citrate if hyperkalemia risk	Potassium citrate raises citrate and also lowers calcium excretion; sodium-based therapies can increase calcium excretion
7. Monitor response and adjust therapy	Reassess 24-hour urine citrate and stone risk profile after intervention	Optimize treatment to achieve citrate >1.66 mmol (320 mg)/day and reduce supersaturation

### Citrate resistance

One challenging condition not uncommonly encountered by practitioners is “citrate resistance.” This is defined by an appropriate increase in urinary potassium but not urinary citrate after ingestion of potassium citrate. This occurs in varying degrees from low citraturic response relative to the kaliuretic response, to zero increase in urinary citrate. The pathogenesis of citrate resistance is not yet known at present.

Approximately 90% of citrate in the human body is held in mineralized tissues, where it plays a critical role in regulation of metabolic functions and maintenance of structural integrity of bones [51]. One potential basis for resistance to therapeutic citrate is sequestration in bone due to some prior state of skeletal citrate deficiency. The low urinary citrate-to-creatinine ratio described in chronic kidney disease likely reflects a state of subtle acid excess or early bone disease in CKD [52].

## CONCLUSION AND FUTURE

Despite the long history of efficacy in prevention of calcium kidney stones, citrate therapy presents both challenges and opportunities in the future. One common sequela of pathogenic hypocitraturia is calcium phosphate stones. Calcium phosphate stone formation is driven mainly by alkalinuria and also low citrate [53]. Therapy with K_3_Citrate is a double-edged sword that can be self-defeating as it raises urine citrate (beneficial) and urine pH (deleterious). Calcium phosphate urolithiasis has remained one of the biggest treatment challenges in kidney stones [53]. Hydroxycitrate, a natural compound derived from plants, inhibits cytoplasmic ATP citrate lyase in the proximal tubule and has the potential to increase urinary citrate excretion without raising urine pH [54]. Currently, hydroxycitrate is available only as an over-the-counter dietary supplement derived from *Garcinia cambogia*, typically in capsule or tablet form. There is no pharmaceutical grade hydroxycitrate available yet. It is not approved for stone prevention, and reported side-effects include gastrointestinal discomfort, headache, and rarely hepatotoxicity. Anthranilic acids are a class of drugs that act as NaDC-1 transporter inhibitors [55]. The previously slow pace of NaDC-1 inhibitor development is now accelerating, driven by recent advances in resolving the protein's structure [56]. Continued research into citrate metabolism and transport mechanisms will be key to advancing prevention strategies for stone disease.

## Data Availability

No new data was generated or analyzed in support of this research.
